# Histopathology of Polymicrogyria

**DOI:** 10.15844/pedneurbriefs-29-11-5

**Published:** 2015-11

**Authors:** Iga Fudyma, Nitin R. Wadhwani

**Affiliations:** 1 Northeastern Illinois University, Chicago, IL; 2 Department of Pathology and Laboratory Medicine, Ann & Robert H. Lurie Children's Hospital of Chicago, Chicago, IL

**Keywords:** Polymicrogyria, Cortical Malformation, Histopathology

## Abstract

Investigators from Sainte Justine Hospital (Montreal), Montreal Neurological Hospital and Institute, King's College Hospital (London), and John Radcliffe Hospital (Oxford) retrospectively reviewed medical records, autopsy reports, and genetic studies containing “Polymicrogyria.”

Investigators from Sainte Justine Hospital (Montreal), Montreal Neurological Hospital and Institute, King's College Hospital (London), and John Radcliffe Hospital (Oxford) retrospectively reviewed medical records, autopsy reports, and genetic studies containing “Polymicrogyria.” When available, etiology was assigned and approximately 23 cases had genetic or presumed genetic causes given the presence of multiple congenital anomalies. Five patients had ischemic changes in the CNS localized to the middle cerebral artery. Ten other patients had hypoxic-ischemic changes in the brain. Infection was found in 6. 32% of patients had no attributed etiology.

The brain surface was abnormal overlying the polymicrogyric cortex in 87% of the cases. The leptomeninges were abnormal in 86% of those patients, some showing glial invasion. The majority of cases in which the leptomeninges were abnormal had either a genetic, ischemic, or infectious etiology. In the cases where the leptomeninges were normal, the underlying pia had pathology in a subset of cases (disruption, thickening, subpial gliosis). The grey white matter junction in the majority of cases was blurred, providing further evidence of abnormal cortical geography. Moreover, features of cortical dysplasia were seen in approximately 60% of the cases. In cases where the leptomeninges were abnormal, there was a greater incidence of heterotopic neurons located deep in the white matter. Global brain malformations were also seen which included partial or complete agenesis of the corpus callosum and hippocampal sclerosis. Abnormalities in the brainstem and cerebellum were also present (hypoplastic pyramidal tracts, olivary and dentate nuclei malformations). [1]

COMMENTARY. The post mortem diagnostic threshold for PMG is low, especially in perinatal cases. The astute clinician will often review perinatal imaging prior to or during the prosection for clinical pathological continuity. It was noted that ventriculomegaly was associated with cortical abnormalities including PMG. Other genetic associations with PMG have been reported in the literature (PI3/Akt) which resulted in concomitant PMG and hemimegalencephaly.

While strictly speaking, etiology was only assigned to one category, there is an overlay of histopathologic features amongst all three categories. While most easily identifiable at low power, abnormalities to the leptomeninges and the underlying cortex may only be clinically significant in the appropriate setting. Abnormal elements of the cortical surface without a historical prodrome of epilepsy, developmental delay, motor problems, etc. may not be clinically relevant as it is quite likely that these subtle abnormalities can be present in the unaffected population. Moreover, it is well known that the fetal meninges provide the scaffolding for corticogenesis and neuronal migration.

The set of patients identified by the authors had a large number of perinatal tissue samples. Normal gyration begins at 21 weeks, which essentially includes a well-developed gyrus rectus, insula, and cingulate gyrus. Development of the parahippocampal and superior temporal gyrus are just beginning, and in some instances the brain is relatively lissencephalic at this stage. Examination of the frontal and parietal lobes is paramount in this age group and the majority of landmarks for regional development occur in these two lobes.

While PMG is considered an isolated abnormality, in clinical practice, it is often associated with aforementioned abnormalities. The leptomeninges overlying the pial surface play an important role in cortical development and the authors highlight it is necessary, but not sufficient alone to cause anomalies seen in patients with PMG. [1, 2]
